# Tris(5-amino-1*H*-1,2,4-triazol-4-ium) dihydrogenphosphate hydrogen­phosphate trihydrate

**DOI:** 10.1107/S1600536812044492

**Published:** 2012-11-03

**Authors:** Mohamed Lahbib Mrad, Matthias Zeller, Kristen J. Hernandez, Mohamed Rzaigui, Cherif Ben Nasr

**Affiliations:** a Laboratoire de Chimie des Matériaux, Faculté des sciences de Bizerte, 7021 Zarzouna, Tunisia; b Youngstown State University, Department of Chemistry, One University Plaza, Youngstown, Ohio 44555-3663, USA

## Abstract

In the crystal structure of the title molecular salt, 3C_2_H_5_N_4_^+^·HPO_4_^2−^·H_2_PO_4_^−^·3H_2_O, the phosphate-based framework is built upon layers parallel to (010) made up from the H_2_PO_4_^−^ and HPO_4_^2−^ anions and water mol­ecules, which are inter­connected through O—H⋯O hydrogen bonds. The organic cations are located between the phosphate–water layers and are connected to them *via* N—H⋯O hydrogen bonds. The bond-length features are consistent with an imino resonance form for the exocyclic amino group, as is commonly found for a C—N single bond involving *sp* ^2^-hybridized C and N atoms.

## Related literature

For applications of organic phosphate complexes, see: Bringley & Rajeswaran (2006 ▶); Dai *et al.* (2002 ▶); Masse *et al.* (1993 ▶). For graph-set motifs and theory, see: Bernstein *et al.* (1995 ▶). For reference structural data, see: Kaabi *et al.* (2004 ▶); Shanmuga Sundara Raj *et al.* (2000 ▶). For P—OH bond lengths, see: Chtioui & Jouini (2005 ▶). 
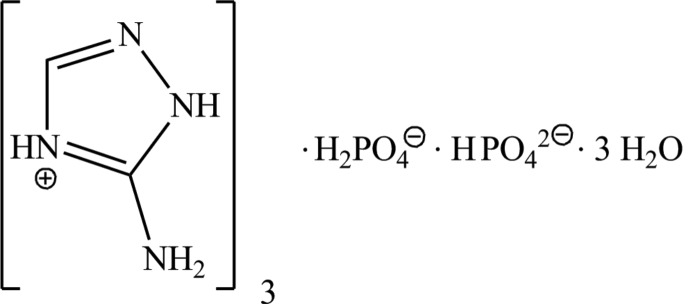


## Experimental

### 

#### Crystal data

3C_2_H_5_N_4_^+^·HO_4_P^2−^·H_2_O_4_P^−^·3H_2_O
*M* *_r_* = 502.31
Monoclinic, 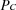

*a* = 10.4793 (13) Å
*b* = 8.7655 (11) Å
*c* = 11.4536 (14) Å
β = 107.489 (2)°
*V* = 1003.5 (2) Å^3^
*Z* = 2
Mo *K*α radiation
μ = 0.30 mm^−1^
*T* = 100 K
0.60 × 0.35 × 0.18 mm

#### Data collection

Bruker SMART APEX CCD diffractometer
Absorption correction: multi-scan (*SADABS*; Bruker, 2011 ▶) *T*_min_ = 0.693, *T*_max_ = 0.746
13833 measured reflections
6229 independent reflections
6132 reflections with *I* > 2σ(*I*)
*R*_int_ = 0.016

#### Refinement

*R*[*F* ^2^ > 2σ(*F* ^2^)] = 0.023
*wR*(*F* ^2^) = 0.059
*S* = 1.04
6229 reflections
322 parameters
32 restraints
H atoms treated by a mixture of independent and constrained refinement
Δρ_max_ = 0.33 e Å^−3^
Δρ_min_ = −0.19 e Å^−3^
Absolute structure: Flack (1983 ▶), 2950 Friedel pairs
Flack parameter: −0.02 (4)

### 

Data collection: *APEX2* (Bruker, 2011 ▶); cell refinement: *SAINT* (Bruker, 2011 ▶); data reduction: *SAINT*; program(s) used to solve structure: *SHELXTL* (Sheldrick, 2008 ▶); program(s) used to refine structure: *SHELXLE* (Hübschle *et al.*, 2011 ▶); molecular graphics: *SHELXTL* (Sheldrick, 2008 ▶); software used to prepare material for publication: *SHELXTL* and *publCIF* (Westrip, 2010 ▶).

## Supplementary Material

Crystal structure: contains datablock(s) global, I. DOI: 10.1107/S1600536812044492/ru2044sup1.cif

Structure factors: contains datablock(s) I. DOI: 10.1107/S1600536812044492/ru2044Isup2.hkl

Supplementary material file. DOI: 10.1107/S1600536812044492/ru2044Isup3.cml

Additional supplementary materials:  crystallographic information; 3D view; checkCIF report

## Figures and Tables

**Table 1:** Hydrogen-bond geometry (Å, °)

*D*—H⋯*A*	*D*—H	H⋯*A*	*D*⋯*A*	*D*—H⋯*A*
N1*A*—H1*A*1⋯O3*B*	0.85 (1)	2.31 (1)	3.1356 (13)	162 (2)
N1*A*—H1*A*2⋯N3*A* ^i^	0.85 (1)	2.19 (1)	3.0305 (15)	170 (2)
N2*A*—H2*A*⋯O4*B*	0.88	1.77	2.6130 (13)	161
N4*A*—H4*A*⋯O4*B* ^i^	0.88	1.76	2.6314 (13)	171
N1*B*—H1*B*1⋯O2	0.86 (1)	1.96 (1)	2.8214 (13)	178 (2)
N1*B*—H1*B*2⋯N3*B* ^i^	0.82 (1)	2.28 (1)	3.0639 (15)	160 (2)
N2*B*—H2*B*1⋯O3^ii^	0.88	1.84	2.6824 (13)	159
N4*B*—H4*B*⋯O1*A* ^iii^	0.88	1.87	2.7376 (12)	167
N1*C*—H1*C*1⋯N3*C* ^i^	0.83 (1)	2.18 (1)	3.0028 (14)	172 (2)
N1*C*—H1*C*2⋯O3*A*	0.86 (1)	2.24 (1)	3.0589 (13)	160 (2)
N2*C*—H2*C*⋯O4*A*	0.88	1.78	2.6278 (12)	161
N4*C*—H4*C*⋯O4*A* ^i^	0.88	1.79	2.6645 (12)	170
O2*A*—H2*AB*⋯O3*B* ^iv^	0.76	1.95	2.6593 (11)	155
O1*B*—H1*B*⋯O3*A*	0.77	1.80	2.5495 (12)	161
O2*B*—H2*BA*⋯O1^iv^	0.83	1.73	2.5552 (12)	176
O1—H1*D*⋯O2	0.84 (1)	1.93 (1)	2.7439 (12)	166 (2)
O1—H1*E*⋯O3*B*	0.80 (1)	1.91 (1)	2.6968 (12)	168 (2)
O2—H2*D*⋯O1*A* ^v^	0.82 (1)	1.90 (1)	2.7024 (11)	168 (2)
O2—H2*E*⋯O3*A* ^iii^	0.81 (1)	1.95 (1)	2.7566 (12)	178 (2)
O3—H3*D*⋯O2*B*	0.79 (1)	2.14 (2)	2.8515 (12)	149 (2)
O3—H3*E*⋯O1*A* ^iii^	0.80 (1)	1.92 (1)	2.7085 (11)	176 (2)

Symmetry codes: (i) 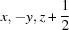; (ii) 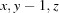; (iii) 
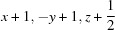
; (iv) 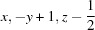; (v) 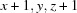.

**Fig. 1.**
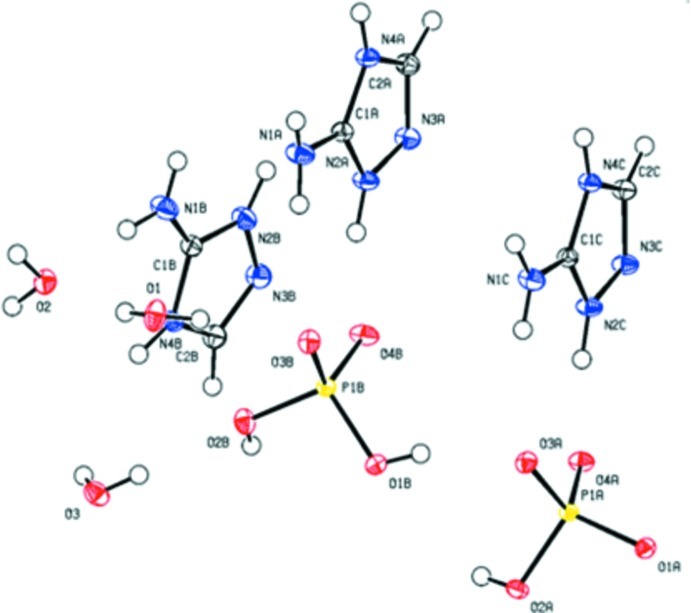
A view of the title compound, showing 40% probability displacement ellipsoids and arbitrary spheres for the H atoms.

**Fig. 2.**
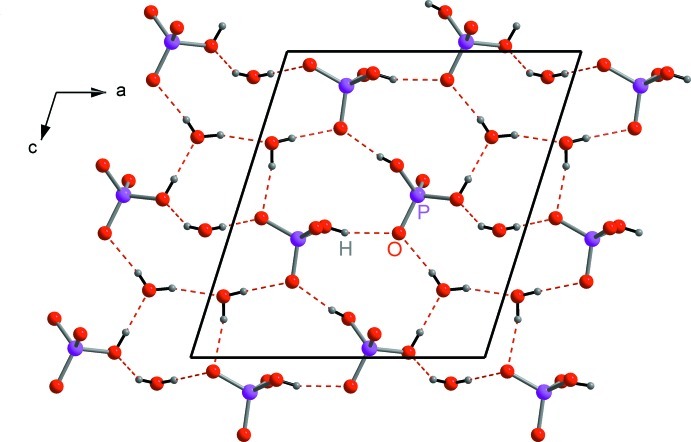
Projection along the b-axis of the inorganic layers in the structure of the title compound. PO4 is given in the tetrahedral representation. Hydrogen bonds are shown as broken lines.

**Fig. 3.**
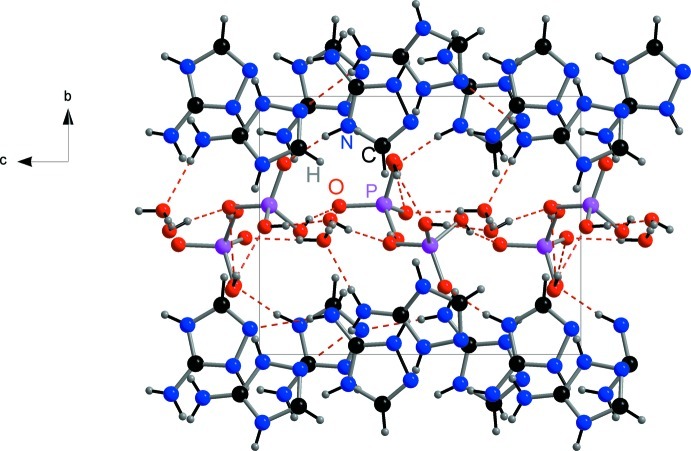
The packing diagram of the compound viewed down the a-axis. PO4 is given in the tetrahedral representation. Hydrogen bonds are shown as broken lines.

**Fig. 4.**
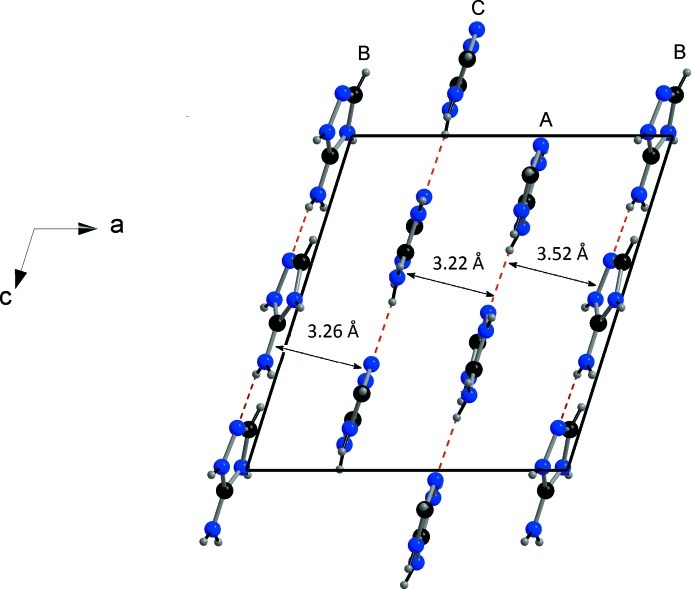
Projection along the b axis of the organic chains in the structure of the title compound. Hydrogen bonds are shown as broken lines. Numbers are interplanar spacings between layers of organic molecules of type A, B and C.

## supplementary crystallographic information

### Comment

Inorganic–organic hybrid compounds provide a class of materials with interesting technological applications (Bringley & Rajeswaran, 2006; Dai *et al.*, 2002). Among these materials, compounds with noncentrosymmetric crystallographic structures are interesting for their applications in quadratic non-linear optical materials research (Masse *et al.*, 1993). Their abilities to combine the rigidity and high cohesion of inorganic host matrices with the enhanced polarizability of organic guest chromophores within one molecular scale assists in better performance of optical signal-processing devices. The use of organic-inorganic polar crystalline materials for quadratic nonlinear optical applications is supported by two observations:

(i) the organic molecules, especially if they contain a delocalized π-system with asymmetric substitution by electron donor-acceptor groups, are highly polarizable entities idealy suited for NLO applications. Being organic materials, the nature of the substituents can be tailored so as to not affect optical transparency;

(ii) the ionic inorganic host matrices are able to increase the packing cohesion, can induce noncentrosymmetry, and also shift the transparency of crystal towards blue wavelengths.

Within a systematic investigation of new materials resulting from the association of organic chromophores with inorganic species, we report here the synthesis and the characterization of a new hybrid phosphate-amine material, (C_2_H_5_N_4_)_3_(HPO_4_)(H_2_PO_4_).3H_2_O, which includes the 3-amino-1*H*-1,2,4-triazolium cations, a chromophore which could be efficient in the blue-U.V. wavelength region. The title compound could exhibit a richness of interesting physical properties such as ferroelectricity and nonlinear optic phenomena like second harmonic generation. It crystallizes in a non-centrosymmetric setting in the space group *Pc*. The structure of this organic-inorganic hybrid material consists of one dihydrogenmonophosphate anion, one monohydrogenmonophosphate dianion, three crystallographically independent 3-amino-1*H*-1,2,4-triazolium cations and three water molecules (Fig. 1). The atomic arrangement is a typical layered organization as it is very often encountered in this kind of inorganic-organic hybrid compounds (Kaabi *et al.*, 2004). The H_2_PO_4_^-^ anions are hydrogen bonded with the HPO_4_^2-^ groups and one of the water molecules (that of O3) to form corrugated chains running parallel to the *a*-axis at (0, 0, 0) and (0, 0, 1/2). These chains are interconnected, *via* O(water)—H···O and O—H···O(water) hydrogen bonds, with the two remaining water molecules H_2_O(1) and H_2_O(2), associated through O1—H···O2 hydrogen bonds, on one hand, and with the HPO_4_^2-^ anions of the adjacent chain, trough O—H···O hydrogen bonds, on the other hand. These hydrogen bonds link the different inorganic units into infinite planar layers parallel to the (0 1 0) plane (Fig. 2) crossing the unit cell at *y* = (2n +1)/2 (Fig. 3). Within the layers, various graph-set motifs (Bernstein *et al.*, 1995) are apparent, including *R*_5_^5^(10) and *R*_4_^4^(12) loops. The 3-amino-1*H*-1,2,4-triazolium cations are interconnected *via* weak N—H···N hydrogen bonds, with D—H···A distances between 3.003 (1) and 3.064 (1) Å, to form organic chains spreading along the *c*-axis at *x* ~ (n + 1)/3 (Fig. 4). The chains are build from the three crystallographically independent organic cations, labelled A, B and C, in such a way that each N—H···N connected chain incorporates only one type of cation: Molecules of type A are located at *x* ~1/3, chains at *x* ~ 0 consist of molecules of type B, and the chains at *x*~2/3 are made up of molecules C. Alternating molecules in each of these chains are created by the c-glide plane. In two of the chains, that of molecules A and C, alternating molecules are roughly coplanar. In the third, molecules are twisted against each other by an angle of 34.37°. The chains are roughly parallel to each other and weakly π-stacked, with interplanar distances between the mean planes of chains between 3.21 Å (between A and C), and up to 3.52 Å (for A and B). Despite of the quite close interplanar distances, π-π stacking interactions are limited due to molecule offsets in parallel layers, and the non-coplanarity of neighboring molecules in the chains of molecules B. The organic chains are anchored to the inorganic layers through N—H···O hydrogen bonds whose geometrical characteristics are given in Table 2. The projection of the whole arrangement along the *a*-axis (Fig. 4) shows how the organic chains alternate as to fill the space separating parallel inorganic layers. In this structure, three 3-amino-1*H*-1,2,4-triazolium cationic groups compensate the negative charges of the dihydrogenmonophosphate and the monohydrogenmonophosphate anions, leading to charge neutrality for the structure as a whole.

The sum of the angles around the N1A, N1B and N1C nitrogen atoms are 360° and the C—N bond distances of the NH_2_ groups are 1.332 (1) Å for N1A—C1A, 1.327 (1) Å for N1B—C1B and 1.330 (1) Å for N1C—C1C, which are short for C—N single bonds, but still not quite as contracted as one would expect for a fully established C=N double bond. These bond length features are consistent with an imino resonance form as it is commonly found for a C—N single bond involving *sp*^2^ hybridized C and N atoms (Shanmuga Sundara Raj *et al.*, 2000). In agreement with this, the amino groups are not pyramidal but the electron densities of the hydrogen atoms of the amino groups were found to be in plane with the 3-amino-1*H*-1,2,4-triazolium skeleton. The detailed geometry of the HP(1 A)O_4_^2-^ and H_2_P(1B)O_4_^-^ anions shows two kinds of P—O distances. The shortest ones, 1.5243 (8), 1.5294 (8) and 1.5364 Å for the first anion (labelled A) and 1.5132 (8) and 1.5163 (8) Å for the second one (labelled B), correspond to the phosphorous atom doubly bonded to the oxygen atom, while the largest ones 1.5845 (8) Å and (1.5612 (8), 1.5741 (8) Å, respectively, can be attributed to the P—OH bond length. This is in agreement with the literature data (Chtioui & Jouini, 2005). Refining the structure in the asymmetric space group gives a value of -0.02 (4) for the Flack parameter (Flack, 1983), confirming the absolute structure and absence of twinning.

### Experimental

Crystals of the title compound were prepared at room temperature by slow addition of a solution of orthophosphoric acid (8 mmol in 30 ml of water) to an alcoholic solution of 3-amino-1*H*-1,2,4-triazole (12 mmol in 30 ml of ethanol). The acid was added until the alcoholic solution became turbid. After filtration, the solution was allowed to slowly evaporate at room temperature over several days leading to formation of transparent prismatic crystals with suitable dimensions for single-crystal structural analysis (1.2 mg, 2.4 mmol, yield 60%). The crystals are stable for months under normal conditions of temperature and humidity.

### Refinement

H atoms were placed in calculated positions with the exception of water and NH_2_ H atoms, which were located in difference density maps and were refined. C—H distances were set to 0.95 Å, N_ring_—H distances to 0.88 Å. H atoms of P-bound hydroxy groups were placed geometrically with fixed P—O—H angles, but with variable torional angles and O—H distances to best fit the experimental electron density (AFIX 148 in *SHELXTL*, Sheldrick 2008). All H_2_O O—H distances were restrained to be similar within a standard deviation of 0.02 Å. All amino N—H distances were also restrained to be similar within the same standard deviation. *U*_iso_ values of H atoms were set to 1.2 or 1.5 times *U*_eq_ of their respective carrier atom for amino and O-bound H atoms respectively.

### Crystal data

**Table:**

3C_2_H_5_N_4_^+^·HO_4_P^2^^−^·H_2_O_4_P^−^·3H_2_O	*F*(000) = 524
*M**_r_* = 502.31	*D*_x_ = 1.662 Mg m^−^^3^
Monoclinic, *P**c*	Mo *K*α radiation, λ = 0.71073 Å
Hall symbol: P -2yc	Cell parameters from 5573 reflections
*a* = 10.4793 (13) Å	θ = 3.0–31.8°
*b* = 8.7655 (11) Å	µ = 0.30 mm^−^^1^
*c* = 11.4536 (14) Å	*T* = 100 K
β = 107.489 (2)°	Block, colourless
*V* = 1003.5 (2) Å^3^	0.60 × 0.35 × 0.18 mm
*Z* = 2	

### Data collection

**Table:**

Bruker SMART APEX CCD diffractometer	6229 independent reflections
Radiation source: fine-focus sealed tube	6132 reflections with *I* > 2σ(*I*)
Graphite monochromator	*R*_int_ = 0.016
ω scans	θ_max_ = 32.0°, θ_min_ = 2.3°
Absorption correction: multi-scan (*SADABS*; Bruker, 2011)	*h* = −14→15
*T*_min_ = 0.693, *T*_max_ = 0.746	*k* = −12→13
13833 measured reflections	*l* = −16→16

### Refinement

**Table:**

Refinement on *F*^2^	Secondary atom site location: difference Fourier map
Least-squares matrix: full	Hydrogen site location: inferred from neighbouring sites
*R*[*F*^2^ > 2σ(*F*^2^)] = 0.023	H atoms treated by a mixture of independent and constrained refinement
*wR*(*F*^2^) = 0.059	*w* = 1/[σ^2^(*F*_o_^2^) + (0.0385*P*)^2^ + 0.0562*P*] where *P* = (*F*_o_^2^ + 2*F*_c_^2^)/3
*S* = 1.04	(Δ/σ)_max_ < 0.001
6229 reflections	Δρ_max_ = 0.33 e Å^−^^3^
322 parameters	Δρ_min_ = −0.19 e Å^−^^3^
32 restraints	Absolute structure: Flack (1983), 2950 Friedel pairs
Primary atom site location: structure-invariant direct methods	Flack parameter: −0.02 (4)

### Special details

**Table:**

Geometry. All e.s.d.'s (except the e.s.d. in the dihedral angle between two l.s. planes) are estimated using the full covariance matrix. The cell e.s.d.'s are taken into account individually in the estimation of e.s.d.'s in distances, angles and torsion angles; correlations between e.s.d.'s in cell parameters are only used when they are defined by crystal symmetry. An approximate (isotropic) treatment of cell e.s.d.'s is used for estimating e.s.d.'s involving l.s. planes.
Refinement. Refinement of *F*^2^ against ALL reflections. The weighted *R*-factor *wR* and goodness of fit *S* are based on *F*^2^, conventional *R*-factors *R* are based on *F*, with *F* set to zero for negative *F*^2^. The threshold expression of *F*^2^ > σ(*F*^2^) is used only for calculating *R*-factors(gt) *etc*. and is not relevant to the choice of reflections for refinement. *R*-factors based on *F*^2^ are statistically about twice as large as those based on *F*, and *R*- factors based on ALL data will be even larger.

### Fractional atomic coordinates and isotropic or equivalent isotropic displacement parameters (Å^2^)

**Table:**

	*x*	*y*	*z*	*U*_iso_*/*U*_eq_	
N1A	0.62032 (11)	0.15272 (11)	0.77449 (9)	0.01772 (18)	
H1A1	0.6049 (17)	0.2392 (16)	0.7386 (16)	0.021*	
H1A2	0.6042 (17)	0.1391 (19)	0.8421 (13)	0.021*	
N2A	0.60906 (10)	0.04376 (11)	0.58172 (9)	0.01488 (17)	
H2A	0.6141	0.1293	0.5432	0.018*	
N3A	0.59896 (10)	−0.09961 (11)	0.52940 (9)	0.01524 (17)	
N4A	0.60216 (9)	−0.11435 (11)	0.72352 (9)	0.01336 (16)	
H4A	0.6017	−0.1543	0.7939	0.016*	
C1A	0.61011 (10)	0.03524 (12)	0.69840 (10)	0.01299 (18)	
C2A	0.59494 (11)	−0.19168 (13)	0.61746 (10)	0.01451 (18)	
H2AA	0.5879	−0.2995	0.6093	0.017*	
N1B	0.95278 (11)	0.16763 (12)	0.67577 (9)	0.01933 (19)	
H1B1	0.9835 (17)	0.2502 (16)	0.7144 (15)	0.023*	
H1B2	0.9369 (18)	0.0936 (17)	0.7129 (16)	0.023*	
N2B	0.92010 (10)	0.02876 (11)	0.48939 (9)	0.01598 (17)	
H2B1	0.8958	−0.0591	0.5132	0.019*	
N3B	0.93026 (11)	0.05748 (11)	0.37327 (10)	0.01706 (18)	
N4B	0.98316 (9)	0.26338 (10)	0.49075 (8)	0.01343 (16)	
H4B	1.0082	0.3574	0.5133	0.016*	
C1B	0.95240 (10)	0.15303 (12)	0.56033 (10)	0.01362 (18)	
C2B	0.96750 (11)	0.19962 (13)	0.37803 (10)	0.01543 (19)	
H2B2	0.9821	0.2531	0.3110	0.019*	
N1C	0.28072 (10)	0.15370 (11)	0.42295 (9)	0.01621 (17)	
H1C1	0.2900 (16)	0.137 (2)	0.4966 (12)	0.019*	
H1C2	0.2796 (17)	0.2424 (15)	0.3916 (15)	0.019*	
N2C	0.28411 (10)	0.04801 (11)	0.23314 (8)	0.01377 (16)	
H2C	0.2843	0.1345	0.1942	0.017*	
N3C	0.28565 (10)	−0.09510 (11)	0.18257 (9)	0.01465 (17)	
N4C	0.28158 (9)	−0.11349 (11)	0.37482 (8)	0.01238 (16)	
H4C	0.2795	−0.1547	0.4443	0.015*	
C1C	0.28234 (10)	0.03715 (12)	0.34900 (9)	0.01169 (17)	
C2C	0.28461 (10)	−0.18892 (12)	0.27069 (10)	0.01394 (18)	
H2CA	0.2858	−0.2969	0.2636	0.017*	
P1A	0.23842 (2)	0.41894 (3)	0.11253 (2)	0.00876 (5)	
O1A	0.09137 (7)	0.45148 (9)	0.04480 (7)	0.01176 (13)	
O2A	0.31520 (8)	0.55501 (9)	0.07195 (7)	0.01273 (14)	
H2AB	0.3902 (19)	0.5380 (10)	0.0918 (15)	0.019*	
O3A	0.26391 (8)	0.42577 (8)	0.25172 (7)	0.01225 (14)	
O4A	0.28676 (8)	0.26682 (9)	0.07706 (7)	0.01314 (14)	
P1B	0.59788 (2)	0.42266 (3)	0.46934 (2)	0.00962 (5)	
O1B	0.49521 (8)	0.52280 (9)	0.37270 (7)	0.01584 (15)	
H1B	0.4300 (18)	0.4769 (15)	0.3450 (15)	0.024*	
O2B	0.73507 (8)	0.50517 (10)	0.48323 (7)	0.01466 (14)	
H2BA	0.7526 (10)	0.5008 (18)	0.4177 (17)	0.022*	
O3B	0.57479 (8)	0.43008 (9)	0.59371 (7)	0.01269 (14)	
O4B	0.59985 (9)	0.26114 (9)	0.42328 (7)	0.01614 (15)	
O1	0.78679 (8)	0.52112 (11)	0.77991 (8)	0.01834 (16)	
H1D	0.8624 (15)	0.502 (2)	0.7733 (18)	0.028*	
H1E	0.7308 (17)	0.484 (2)	0.7240 (15)	0.028*	
O2	1.04601 (8)	0.44288 (10)	0.79982 (7)	0.01506 (15)	
H2D	1.0714 (18)	0.4441 (19)	0.8747 (12)	0.023*	
H2E	1.1091 (15)	0.481 (2)	0.7840 (17)	0.023*	
O3	0.91292 (8)	0.75025 (9)	0.58426 (8)	0.01633 (15)	
H3D	0.8480 (15)	0.699 (2)	0.5712 (17)	0.025*	
H3E	0.9680 (16)	0.6944 (19)	0.5732 (17)	0.025*	

### Atomic displacement parameters (Å^2^)

**Table:**

	*U*^11^	*U*^22^	*U*^33^	*U*^12^	*U*^13^	*U*^23^
N1A	0.0286 (5)	0.0117 (4)	0.0144 (4)	−0.0010 (4)	0.0086 (4)	−0.0005 (3)
N2A	0.0231 (4)	0.0110 (4)	0.0115 (4)	−0.0008 (3)	0.0067 (3)	0.0012 (3)
N3A	0.0212 (4)	0.0128 (4)	0.0127 (4)	−0.0002 (3)	0.0067 (3)	−0.0007 (3)
N4A	0.0176 (4)	0.0118 (4)	0.0114 (4)	0.0000 (3)	0.0054 (3)	0.0021 (3)
C1A	0.0142 (4)	0.0125 (5)	0.0123 (5)	−0.0003 (3)	0.0041 (3)	0.0017 (3)
C2A	0.0177 (4)	0.0134 (5)	0.0134 (5)	−0.0004 (4)	0.0061 (4)	0.0002 (3)
N1B	0.0291 (5)	0.0157 (4)	0.0145 (4)	−0.0063 (4)	0.0085 (4)	−0.0010 (3)
N2B	0.0207 (4)	0.0123 (4)	0.0155 (4)	−0.0025 (3)	0.0063 (3)	−0.0011 (3)
N3B	0.0207 (4)	0.0154 (4)	0.0154 (4)	−0.0004 (3)	0.0058 (3)	−0.0014 (3)
N4B	0.0169 (4)	0.0105 (4)	0.0129 (4)	−0.0015 (3)	0.0045 (3)	−0.0001 (3)
C1B	0.0139 (4)	0.0119 (4)	0.0146 (5)	−0.0004 (3)	0.0037 (3)	0.0000 (3)
C2B	0.0172 (4)	0.0154 (5)	0.0139 (5)	−0.0002 (4)	0.0048 (4)	−0.0006 (4)
N1C	0.0248 (4)	0.0124 (4)	0.0129 (4)	−0.0016 (3)	0.0079 (3)	−0.0013 (3)
N2C	0.0222 (4)	0.0099 (4)	0.0106 (4)	0.0010 (3)	0.0070 (3)	0.0005 (3)
N3C	0.0225 (4)	0.0101 (4)	0.0123 (4)	0.0017 (3)	0.0066 (3)	0.0002 (3)
N4C	0.0167 (4)	0.0104 (4)	0.0109 (4)	−0.0008 (3)	0.0054 (3)	0.0008 (3)
C1C	0.0135 (4)	0.0112 (5)	0.0107 (4)	−0.0004 (3)	0.0040 (3)	0.0008 (3)
C2C	0.0186 (5)	0.0113 (4)	0.0126 (4)	0.0005 (4)	0.0056 (4)	0.0006 (3)
P1A	0.01123 (10)	0.00815 (11)	0.00727 (11)	0.00019 (8)	0.00335 (8)	0.00021 (8)
O1A	0.0118 (3)	0.0126 (3)	0.0107 (3)	0.0004 (3)	0.0031 (2)	0.0007 (3)
O2A	0.0126 (3)	0.0103 (3)	0.0161 (4)	0.0001 (3)	0.0057 (3)	0.0030 (3)
O3A	0.0152 (3)	0.0137 (3)	0.0078 (3)	0.0001 (3)	0.0034 (3)	−0.0008 (2)
O4A	0.0200 (3)	0.0094 (3)	0.0113 (3)	0.0015 (3)	0.0067 (3)	−0.0003 (3)
P1B	0.01187 (10)	0.00900 (11)	0.00812 (11)	−0.00035 (8)	0.00319 (8)	−0.00054 (8)
O1B	0.0154 (3)	0.0138 (4)	0.0150 (4)	−0.0008 (3)	−0.0006 (3)	0.0036 (3)
O2B	0.0131 (3)	0.0193 (4)	0.0127 (3)	−0.0036 (3)	0.0057 (3)	−0.0023 (3)
O3B	0.0136 (3)	0.0160 (4)	0.0095 (3)	−0.0003 (3)	0.0051 (3)	−0.0022 (3)
O4B	0.0291 (4)	0.0099 (3)	0.0115 (3)	−0.0003 (3)	0.0093 (3)	−0.0011 (3)
O1	0.0131 (3)	0.0292 (5)	0.0127 (4)	−0.0018 (3)	0.0039 (3)	−0.0045 (3)
O2	0.0152 (3)	0.0208 (4)	0.0099 (3)	−0.0028 (3)	0.0047 (3)	−0.0007 (3)
O3	0.0162 (3)	0.0120 (4)	0.0225 (4)	−0.0001 (3)	0.0083 (3)	−0.0010 (3)

### Geometric parameters (Å, º)

**Table:**

N1A—C1A	1.3325 (14)	N2C—C1C	1.3361 (13)
N1A—H1A1	0.854 (13)	N2C—N3C	1.3838 (13)
N1A—H1A2	0.849 (13)	N2C—H2C	0.8800
N2A—C1A	1.3353 (14)	N3C—C2C	1.3044 (14)
N2A—N3A	1.3826 (13)	N4C—C1C	1.3537 (14)
N2A—H2A	0.8800	N4C—C2C	1.3724 (14)
N3A—C2A	1.3022 (14)	N4C—H4C	0.8800
N4A—C1A	1.3504 (15)	C2C—H2CA	0.9500
N4A—C2A	1.3732 (14)	P1A—O4A	1.5243 (8)
N4A—H4A	0.8800	P1A—O1A	1.5294 (8)
C2A—H2AA	0.9500	P1A—O3A	1.5364 (8)
N1B—C1B	1.3272 (15)	P1A—O2A	1.5845 (8)
N1B—H1B1	0.859 (13)	O2A—H2AB	0.7639
N1B—H1B2	0.820 (13)	P1B—O4B	1.5132 (8)
N2B—C1B	1.3401 (14)	P1B—O3B	1.5163 (8)
N2B—N3B	1.3888 (14)	P1B—O1B	1.5612 (8)
N2B—H2B1	0.8800	P1B—O2B	1.5741 (8)
N3B—C2B	1.3018 (15)	O1B—H1B	0.7742
N4B—C1B	1.3524 (14)	O2B—H2BA	0.8262
N4B—C2B	1.3706 (14)	O1—H1D	0.835 (14)
N4B—H4B	0.8800	O1—H1E	0.796 (14)
C2B—H2B2	0.9500	O2—H2D	0.819 (13)
N1C—C1C	1.3304 (14)	O2—H2E	0.808 (13)
N1C—H1C1	0.833 (13)	O3—H3D	0.790 (13)
N1C—H1C2	0.855 (13)	O3—H3E	0.795 (13)
			
C1A—N1A—H1A1	113.9 (12)	C1C—N1C—H1C2	115.6 (11)
C1A—N1A—H1A2	119.3 (12)	H1C1—N1C—H1C2	124.9 (16)
H1A1—N1A—H1A2	120.2 (17)	C1C—N2C—N3C	110.88 (9)
C1A—N2A—N3A	111.07 (9)	C1C—N2C—H2C	124.6
C1A—N2A—H2A	124.5	N3C—N2C—H2C	124.6
N3A—N2A—H2A	124.5	C2C—N3C—N2C	104.11 (9)
C2A—N3A—N2A	104.11 (9)	C1C—N4C—C2C	106.08 (9)
C1A—N4A—C2A	106.34 (9)	C1C—N4C—H4C	127.0
C1A—N4A—H4A	126.8	C2C—N4C—H4C	127.0
C2A—N4A—H4A	126.8	N1C—C1C—N2C	125.74 (10)
N1A—C1A—N2A	125.90 (10)	N1C—C1C—N4C	127.44 (10)
N1A—C1A—N4A	127.52 (10)	N2C—C1C—N4C	106.81 (9)
N2A—C1A—N4A	106.56 (9)	N3C—C2C—N4C	112.11 (10)
N3A—C2A—N4A	111.92 (10)	N3C—C2C—H2CA	123.9
N3A—C2A—H2AA	124.0	N4C—C2C—H2CA	123.9
N4A—C2A—H2AA	124.0	O4A—P1A—O1A	113.13 (4)
C1B—N1B—H1B1	118.9 (12)	O4A—P1A—O3A	110.03 (4)
C1B—N1B—H1B2	120.0 (13)	O1A—P1A—O3A	110.70 (4)
H1B1—N1B—H1B2	120.2 (18)	O4A—P1A—O2A	109.97 (5)
C1B—N2B—N3B	110.80 (9)	O1A—P1A—O2A	103.52 (4)
C1B—N2B—H2B1	124.6	O3A—P1A—O2A	109.26 (4)
N3B—N2B—H2B1	124.6	P1A—O2A—H2AB	109.5
C2B—N3B—N2B	103.98 (9)	O4B—P1B—O3B	112.98 (5)
C1B—N4B—C2B	106.33 (9)	O4B—P1B—O1B	110.95 (5)
C1B—N4B—H4B	126.8	O3B—P1B—O1B	111.80 (5)
C2B—N4B—H4B	126.8	O4B—P1B—O2B	110.94 (5)
N1B—C1B—N2B	127.37 (10)	O3B—P1B—O2B	106.50 (4)
N1B—C1B—N4B	126.03 (10)	O1B—P1B—O2B	103.13 (5)
N2B—C1B—N4B	106.60 (10)	P1B—O1B—H1B	109.5
N3B—C2B—N4B	112.29 (10)	P1B—O2B—H2BA	109.5
N3B—C2B—H2B2	123.9	H1D—O1—H1E	109.7 (19)
N4B—C2B—H2B2	123.9	H2D—O2—H2E	101.5 (17)
C1C—N1C—H1C1	119.1 (12)	H3D—O3—H3E	104.3 (18)

### Hydrogen-bond geometry (Å, º)

**Table:**

*D*—H···*A*	*D*—H	H···*A*	*D*···*A*	*D*—H···*A*
N1*A*—H1*A*1···O3*B*	0.85 (1)	2.31 (1)	3.1356 (13)	162 (2)
N1*A*—H1*A*2···N3*A*^i^	0.85 (1)	2.19 (1)	3.0305 (15)	170 (2)
N2*A*—H2*A*···O4*B*	0.88	1.77	2.6130 (13)	161
N4*A*—H4*A*···O4*B*^i^	0.88	1.76	2.6314 (13)	171
N1*B*—H1*B*1···O2	0.86 (1)	1.96 (1)	2.8214 (13)	178 (2)
N1*B*—H1*B*2···N3*B*^i^	0.82 (1)	2.28 (1)	3.0639 (15)	160 (2)
N2*B*—H2*B*1···O3^ii^	0.88	1.84	2.6824 (13)	159
N4*B*—H4*B*···O1*A*^iii^	0.88	1.87	2.7376 (12)	167
N1*C*—H1*C*1···N3*C*^i^	0.83 (1)	2.18 (1)	3.0028 (14)	172 (2)
N1*C*—H1*C*2···O3*A*	0.86 (1)	2.24 (1)	3.0589 (13)	160 (2)
N2*C*—H2*C*···O4*A*	0.88	1.78	2.6278 (12)	161
N4*C*—H4*C*···O4*A*^i^	0.88	1.79	2.6645 (12)	170
O2*A*—H2*AB*···O3*B*^iv^	0.76	1.95	2.6593 (11)	155
O1*B*—H1*B*···O3*A*	0.77	1.80	2.5495 (12)	161
O2*B*—H2*BA*···O1^iv^	0.83	1.73	2.5552 (12)	176
O1—H1*D*···O2	0.84 (1)	1.93 (1)	2.7439 (12)	166 (2)
O1—H1*E*···O3*B*	0.80 (1)	1.91 (1)	2.6968 (12)	168 (2)
O2—H2*D*···O1*A*^v^	0.82 (1)	1.90 (1)	2.7024 (11)	168 (2)
O2—H2*E*···O3*A*^iii^	0.81 (1)	1.95 (1)	2.7566 (12)	178 (2)
O3—H3*D*···O2*B*	0.79 (1)	2.14 (2)	2.8515 (12)	149 (2)
O3—H3*E*···O1*A*^iii^	0.80 (1)	1.92 (1)	2.7085 (11)	176 (2)

Symmetry codes: (i) *x*, −*y*, *z*+1/2; (ii) *x*, *y*−1, *z*; (iii) *x*+1, −*y*+1, *z*+1/2; (iv) *x*, −*y*+1, *z*−1/2; (v) *x*+1, *y*, *z*+1.
